# Dataset of producing and curing concrete using domestic treated wastewater

**DOI:** 10.1016/j.dib.2015.12.020

**Published:** 2015-12-17

**Authors:** Gholamreza Asadollahfardi, Mohammad Delnavaz, Vahid Rashnoiee, Alireza Fazeli, Navid Gonabadi

**Affiliations:** a Civil engineering department, Kharazmi University, Tehran, Iran; b Vancouver Community College, Vancouver, BC, Canada; c Iran Novid Azma Laboratory, Tehran, Iran

## Abstract

We tested the setting time of cement, slump and compressive and tensile strength of 54 triplicate cubic samples and 9 cylindrical samples of concrete with and without a Super plasticizer admixture. We produced concrete samples made with drinking water and treated domestic wastewater containing 300, 400 kg/m^3^ of cement before chlorination and then cured concrete samples made with drinking water and treated wastewater. Second, concrete samples made with 350 kg/m^3^ of cement with a Superplasticizer admixture made with drinking water and treated wastewater and then cured with treated wastewater. The compressive strength of all the concrete samples made with treated wastewater had a high coefficient of determination with the control concrete samples. A 28-day tensile strength of all the samples was 96–100% of the tensile strength of the control samples and the setting time was reduced by 30 min which was consistent with a ASTMC191 standard. All samples produced and cured with treated waste water did not have a significant effect on water absorption, slump and surface electrical resistivity tests. However, compressive strength at 21 days of concrete samples using 300 kg/m^3^ of cement in rapid freezing and thawing conditions was about 11% lower than concrete samples made with drinking water.

**Specification Table**

**Table:** 

Subject area	Construction and environment
More specific subject area	Construction material and wastewater reuse
Types of data	Tables, figures and text files
Data format	Raw, filtered and analyzed
How the data were acquired	Scanning electron microscope (SEM), energy-dispersive X-ray spectroscopy (EDX)
Experimental factor	The effect of using treated wastewater on the characteristics of concrete
Experimental features	Using EDX to find percentage of most elements in both concrete samples which made with drinking water and treated wastewater
Data source location	Khoramabad City, Iran, latitude 33.488° and longitude 48.335°
Data accessibility	The data presented in this article and is related to the research paper

## 1 The value of data

• The data indicate the suitability of treated domestic wastewater for producing concrete.
• The data illustrate the initial setting time of cement made with treated domestic wastewater is higher than the cement made with drinking water.
• A high coefficient of determination exists between the compressive strength data of concrete produced with drinking water and concrete produced by the treated domestic wastewater.
• The water absorption and surface electrical resistivity data of the concrete samples made with treated domestic wastewater and drinking water had approximately similar results.
• The compressive strength of concrete samples, under rapid freezing and thawing, with 300 kg/m^3^ of cement which made with treated wastewater at 21 days was 10.11% lower than concrete samples made of drinking water

## 2 Data 1

The strength and durability of concrete is very dependent on the chemical characteristics of cement. According to ACI 201 [1] concrete durability containing Portland cement depends on its ability to resist weathering action, chemical attacks, abrasion or any process which causes damage to concrete. The type 2 Portland cement produced by the Lorstant cement factory was selected and tested using the ASTM-C150 (2004) standard [2]. Table 1 presents the chemical characteristics of type 2 Portland cement. Table 2 indicates physical and chemical characteristic of the domestic sewage before and after treatment.

A300, A350 and A400 labels were used to indicate the concrete samples produced and cured by drinking water as the control samples, and 300, 350 and 400 indicate the kg/m^3^ of cement in one cubic meter of concrete. B300, B350, B400 labels were used for the concrete samples produced with drinking water and cured with treated wastewater. C300, C350 and C400 labels were used to illustrate the concrete samples produced and cured with treated wastewater. Table 3 indicates the design details of different types of the concrete samples in our study.

Table 4 illustrates the results of slump tests. As presented in the table, the workability of concrete made with treated wastewater did not change significantly, compared with concrete made with drinking water.

Table 5 presents the results of concrete water absorption, which indicates concrete permeability. The volume of water adsorption indicates an existing void in the concrete [3], [4]. Reduction of water adsorption causes a decrease in harmful substances moving into the concrete and reduces corrosion [3], [4]. The results of water absorption tests for different concrete samples made with drinking water or treated wastewater were between 2.1% and 3.1%. These results meet BS 1881, PART 122 [5] which state that water absorption should be between 2% and 5%.

Table 6 indicates the results of concrete electrical resistivity tests in 90 days. As presented in Table 6, concrete made with treated wastewater did not affect the concrete electrical resistivity results significantly. The difference between concrete samples produced with treated water and drinking water was from −3.4 to +5.

Table 7 illustrates the results of tensile strength of concrete samples made and cured by drinking water and treated wastewater in 28 days. A 28-day tensile strength of all samples was 96–100% of the tensile strength of the control samples. As presented in Table 5, using treated waste water did not affect tensile strength significantly.

As indicated in Table 8, the compressive strength of concrete samples with 300 kg/m^3^ of cement which made with treated wastewater at 21 days was 10.11% lower than concrete samples made of drinking water. For concrete samples with 350 kg/m^3^ of cement without using microsilica, the compressive strength at 21 days for concrete made with the treated wastewater was 11.7% lower than concrete samples made with drinking water.

## 3 Data 2

**Fig. 1:**
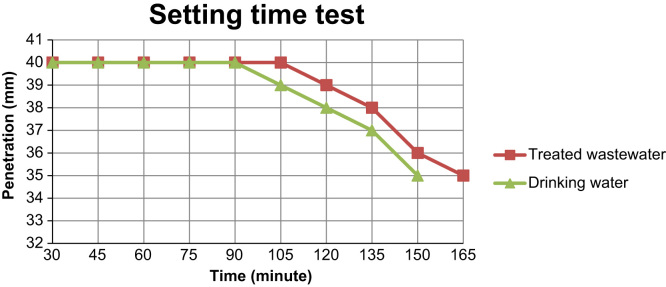
The results of setting time tests using drinking water and treated waste water.

**Fig. 2:**
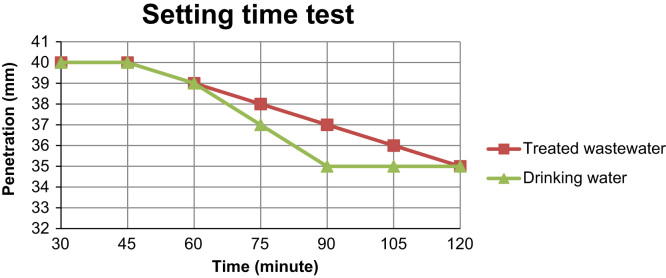
The results of setting time tests using drinking water and treated waste water.

Fig. 1, Fig. 2 illustrate time setting test results which were made by using drinking water and treated wastewater from the Lorstan province wastewater treatment plant and the Ekbatan waste water treatment plant in Iran.

As illustrate in Figs. 1 and 2, the initial time setting using treated waste water increased when compared to using drinking water. Final setting time for sample was made with drinking was 180 min while for samples made with treated wastewater was 240 min.The final setting time for the samples which was made with treated waste also increased.

**Fig. 3:**
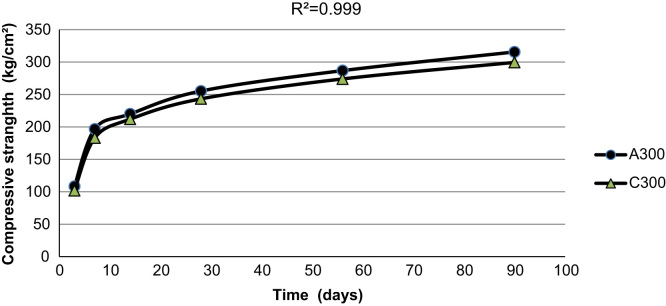
The compressive strength of two concrete samples made with 300 kg/m^3^ of cement using drinking water and treated wastewater.

**Fig. 4:**
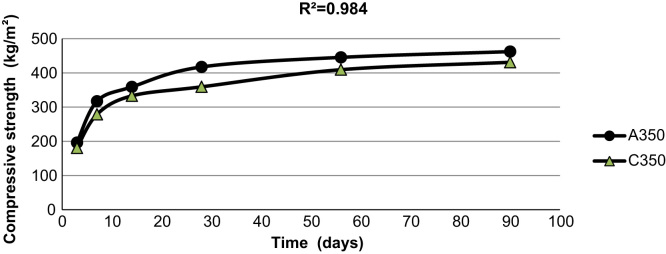
The compressive strength of two concrete samples made with 350 kg/m^3^ of cement with a Super plasticizers admixture using drinking water and treated wastewater.

**Fig. 5:**
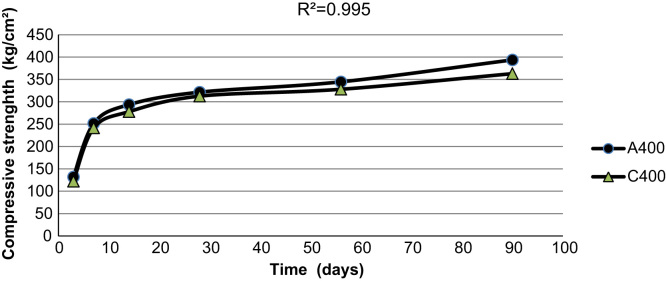
The compressive strength of two concrete samples made with 400 kg/m^3^ of cement using drinking water and treated wastewater.

Fig. 3, Fig. 4, Fig. 5 indicate the compressive strength of concrete samples at 300 kg/m^3^ and 400 kg/m^3^ of cement without Super plasticizer admixtures and 350 kg/m^3^ of cement with an added Super plasticizer admixture. As indicated in the figures, a high coefficient of determinations exists among the various types of concrete samples that were made and cured with treated wastewater and drinking water.

**Fig. 6:**
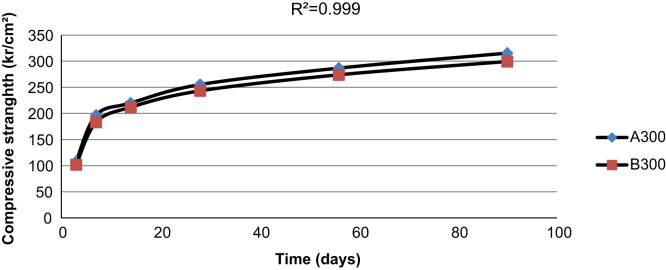
The compressive strength of concrete samples made with 300 kg/m^3^ of cement using drinking water and cured with both drinking water and treated waste water.

**Fig. 7:**
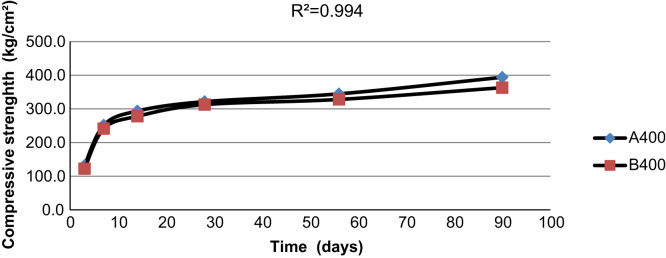
The compressive strength of concrete samples made with 400 kg/m^3^ of cement using drinking water and cured with both drinking water and treated waste water.

Fig. 6, Fig. 7 illustrate the strength of two types of concrete samples made with 300 kg/m^3^ and 400 kg/m^3^ of cement using drinking water and cured with treated wastewater, respectively. In the figures, using treated wastewater for curing concrete samples made with drinking water did not affect the strength of the concrete samples when compared with concrete samples made and cured with drinking water.

**Fig. 8:**
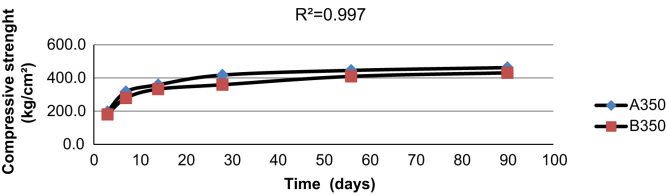
The compressive strength of concrete samples made with 350 kg/m^3^ of cement and a Super plasticizer admixture using drinking water and cured with both drinking water and treated waste water.

Fig. 8 indicates the compressive strength of concrete samples made with 350 kg/m^3^ of cement and a super plasticizer admixture using drinking water and cured with both drinking water and treated waste water. We achieved the same results with concrete made with 300 and 400 kg/m^3^ of cement without a Super plasticizer admixture and concrete made with 350 kg/m^3^ of cement with a Super plasticizers admixture.

**Fig. 9:**
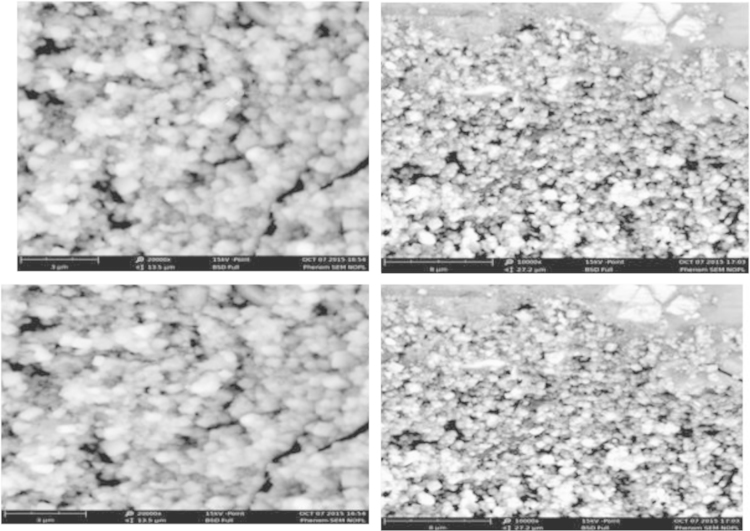
The SEM test for concrete samples made with 300 kg/m^3^ of cement using drinking water and treated wastewater.

**Fig. 10:**
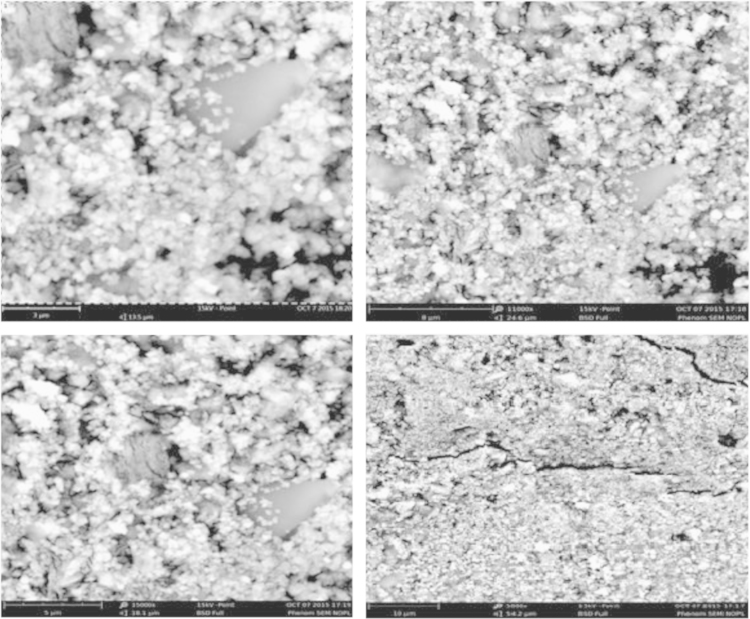
The SEM test for concrete samples made with 300 kg/m^3^ of cement using treated wastewater.

Fig. 9, Fig. 10 indicate the SEM test for concrete samples made with 300 kg/m^3^ of cement using drinking water and treated wastewater, respectively.

The Scanning Electron Microscopy (SEM) images of concrete sample with 300 kg/m^3^ of cement(B300) which was made of treated wastewater (Fig. 9, Fig. 10) present concrete forming of Euhedral crystals. The void between crystals was more than concrete, which was made of drinking water. However, the SEM images of section of concrete sample (Fig. 9, Fig. 10) with 300 kg/m^3^ of cement (A300) which made of drinking water illustrates concrete forming of subhedral to anhedral crystals and more dense and less void than concrete made with treated waste water.

**Fig. 11:**
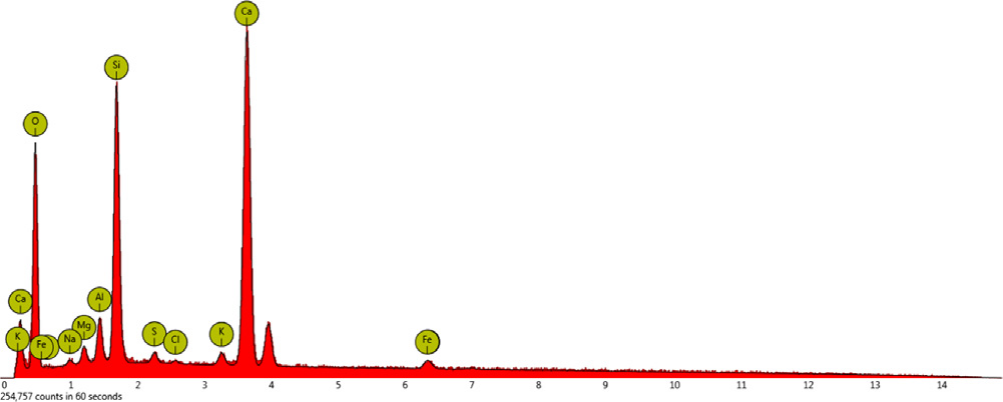
The result of an EDX test of concrete with 300 kg/m^3^ of cement using drinking water.

**Fig. 12:**
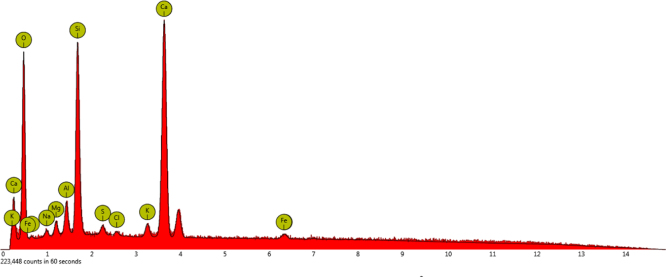
The result of an EDX test of concrete with 300 kg/m^3^ of cement using treated wastewater.

Fig. 11, Fig. 12 indicate the percentage of element in concrete samples made with 300 kg/m^3^ of cement using drinking water and treated wastewater.

As indicated in Fig. 12, the amounts of a few elements such as sodium,chlorine and sulfur in concrete made with treated wastewater were increased compared to concrete using drinking water.

## 4 Material and methods

We used effluent from the Lorstan domestic wastewater treatment plant to produce concrete samples. We examined the physical and chemical characteristics of treated wastewater, Portland cement specification, particle size analysis, cement setting time, slump, compressive strength, tensile strength, concrete water adsorption, surface electrical resistivity, resistance of concrete to rapid freezing and thawing and Scanning Electron Microscopy (SEM) combing energy-dispersive X-ray spectroscopy (EDX). The number of concrete samples produced was 54 triplicate cubic and 9 cylindrical samples with and without a Super plasticizer admixture for compressive and tensile strength tests. Table 9 illustrates the method of examination of all experiments.

## Figures and Tables

**Table 1:** Type 2 Portland cement specifications used in this study.

Chemical compounds of type 2 Portland cement	Maximum and minimum permissible	Testing results
Silicon Dioxide (SiO_2_)	20 (minimum)	21.55
Aluminum Oxide (Al_2_O_3_)	6 (maximum)	5
Ferric Oxide (Fe_2_O_3_)	6 (maximum)	4.3
Magnesium Oxide (MgO)	5 (maximum)	1.78
Sulfur TriOxide (SO_3_)	3 (maximum) 3	2.09
Loss on Ignition (LOI)	3 (maximum)	1.08
Insoluble Residue (I.R)	0.75 (maximum)	0.5
Fineness (Blaine Test, cm^2^/gr)	2800 (minimum)	3000

**Table 2:** Results of testing of domestic sewage before and after treatment the physical and chemical characteristics of raw waste water and treated wastewater.

Parameters	Unit	Influent of aeration lagoons	Effluent of aeration lagoon

Temperature	Celsius	17	17
pH		7.7	7.7
Turbidity	Nephelometric Turbidity Units (NTU)	12	12
Total dissolved solid	Mg/l	420	170
BOD_5_	Mg/l	256	33
Total suspended solid	Mg/l	145	30
Chemical oxygen demand (COD)	Mg/l	432	93

**Table 3:** The design details of different types of concrete samples.

The type of concrete samples	Water/Cement	Cement (Kg)	Free water (Kg)	Coarse Sand (Kg)	Fine sand (Kg)	Gravel (Kg)	Superplasticizer admixture % Structure 335
A300, B300, C300	0.6	300	180	797	212	900	–
A350, B350, C350	0.43	350	150	818	214	880	4
A400, B400, C400	0.5	400	200	734	200	780	

**Table 4:** Slump test results.

Water	Slump (mm)
A300	110
C300	99
A400	90
C400	82
B350	117
C350	105

**Table 5:** The results of concrete water adsorption.

Types of concrete samples	Concrete sample dimensions	Concrete samples cross section (cm^2^)	Concrete samples weight after drying (g)	Weight of concrete samples after setting one hour in water (g)	Water adsorption (%)

A300	15*15*15	225	7760	7985	2.9
B300	15*15*15	225	7765	7995	3
C300	15*15*15	225	7745	7970	3
A350	15*15*15	225	7760	7920	2.1
B350	15*15*15	225	7762	7929	2.2
C350	15*15*15	225	7755	7940	2.4
A400	15*15*15	225	7772	7970	2.6
B400	15*15*15	225	7775	7980	2.7
C400	15*15*15	225	7778	7970	2.6

**Table 6:** The results of concrete electrical resistivity tests in 90 days.

Types of concrete samples	Concrete sample dimensions	Concrete samples cross section (cm^2^)	Concrete electrical resistivity (Ω m)	Difference

A300	15*15*15	225	58	–
B300	15*15*15	225	56	−3.4
C300	15*15*15	225	61	+5
A350	15*15*15	225	51.7	–
B350	15*15*15	225	50	−3
C350	15*15*15	225	55.5	+7
A400	15*15*15	225	73.8	–
B400	15*15*15	225	72	−2.4
C400	15*15*15	225	77	+4

**Table 7:** The results of tensile strength.

Tensile strength (kg/cm^2^)								
A300	B300	C300	A350	B350	C350	A400	B350	C400
21.6	21	22	28	28	27	24.5	24	23.8

**Table 8:** The results of resistance of concrete to rapid freezing and thawing according to ASTM C666/C666M [6].

Types of concrete	Weight (g)	Density (g/cm^3^)	Force (kgf)	Compressive strength (kg/cm^2^)
A300	8130	2.4	60,000	267
B300	8100	2.4	54,000	240
A350 (without microsilica)	7840	2.32	77,000	342
B350 (without microsilica)	7710	2.28	68,000	302

**Table 9:** Method of examination of all the experiments in our study.

Type of testing	Standards
Physical and chemical characteristics of treated wastewater	APHA [7]
Portland cement specification	ASTM-C150 [2]
Standard test method for the sieve analysis of fine and coarse aggregates, Standard specifications of concrete aggregates	ASTM-C136/136M [8] ASTM-C33 [9]
Cement setting time	ASTM-C191 [10]
Concrete slump	ASTM C143 [11]
Compressive strength	BS1881-108 [12]
Tensile strength	BS1881-117 [13]
Concrete water absorption	BS1881-122 [5]
Surface electrical resistivity	FM-5-578 [14]
Resistance of concrete to rapid freezing and thawing	ASTM 666/C666M [6]
Scanning Electron Microscopy (SEM) energy-dispersive X-ray spectroscopy (EDX)	ASTM C1723-10 [15]
